# Adhesive Plaster as a Means of Making Extension in the Compound and Other Fractures of the Lower Extremity

**Published:** 1853-03

**Authors:** 


*Adhesive Plaster as a means of making Extension in the Compound and other Fractures. of the Lower Extremity.* By S. D. GROSS, M. D., Professor of Surgery in the University of Louisville.

UNIVERSITY OF LOUISVILLE, Sept. 27, 1852.

*Gentlemen :*—Will you be so kind as to grant me a little space in your valuable Journal for a few remarks on the subject of Adhesive Plaster, as a means of making extension in compound and other fractures of the inferior extremity? I am induced to ask this favor, because the origin of this mode of treatment, which has lately attracted considerable attention, and which has been adopted with great advantage, in several sections of our country, has been ascribed to a gentleman who is in nowise entitled to the credit of it, if credit it deserves.

In my work on the "Anatomy, Physiology, and Diseases of the Bones and Joints," composed within a few months after I was invested with the honors of the Doctorate, and published in Philadelphia in the summer of 1830, is the following passage:

"In complicated fractures of the leg, it not unfrequently happens that the soft parts about the ankle are so much contused, or otherwise injured, as to render it impossible to employ the usual extending bands. When this is found to be the case, the difficulty may usually be remedied by applying along each side of the leg, as high up as the seat of the fracture will admit, a piece of strong muslin, about two feet and a half in length, two inches and a half in width, and spread at one of its extremities with adhesive plaster. The part which is applied upon the limb should be confined by three or four circular strips, so as to keep it firmly in its place, and equalize the extending power. The free extremities of the extending hands should then be tied under the sole of the foot, and be secured to the block or bar which connects the lower ends of the splints. This mode of making extension, for which we are indebted to the ingenuity of my friend and preceptor, Dr. Swift, of this place, will, I am fully persuaded, be found highly useful in practice, and satisfactorily obviate the inconveniences to which I have alluded."

At the time of writing the work here quoted, I was spending a few months at Easton, Pennsylvania, where I had an opportunity of witnessing the excellent effects of this mode of management. Since that period I have omitted no opportunity of employing it in my own practice; and I have never failed, during the last thirteen years, to speak of it prominently before my classes in the University of Louisville.

Dr. Sargent, in his excellent little work on "Bandaging and other Operations of Minor Surgery," ascribes the credit of this method of extension to Dr. E. Wallace, of Philadelphia; and the same statement is reiterated in that gentleman's edition of Mr. Druitt's Surgery, published at Philadelphia in 1848.

Within the last two years, Dr. Josiah Crosby, of New Hampshire, has published a short account of this mode of treatment, in the New Hampshire Journal of Medicine, illustrated by several cases, in which it appears to have been adopted with the happiest effect. In one of them, a compound fracture of the tibia and fibula, the counter-extending band was applied to the upper part of the leg, and the extending band to the lower part of the leg and foot; the plan answered most admirably, and caused not the slightest inconvenience to the patient. Dr. Crosby states that he has healed two cases of fracture of the clavicle in children two years of age, with nothing but adhesive strips, with as good success as he had ever with the old methods, and with half the trouble. The same mode of treatment has been lately employed with great success in the New York Hospital, in fractures of the inferior extremities.

My conviction is that this plan of making extension deserves to be much more extensively employed than it has hitherto been by my professional brethren. It is particularly applicable to compound and complicated fractures of the leg, but it may also be advantageously resorted to in all cases of fracture of the leg and thigh, in which, on account of injury, excoriation, disease, or excessive morbid sensibility of the ankle, heel, or instep, it is impossible to use the ordinary extending means.

The limb should always, as a matter of course, be shaved before the bands are applied; and the substance of which these bands are composed should be of the most pliant and unyielding character. The adhesive plaster should also be of a very superior quality. The circular strips should not completely encircle the limb, lest they impede the return of the venous blood, and the leg should be carefully bandaged from the toes up, as in the ordinary mode of treatment.

I am, gentlemen, very respectfully,
Your friend and obedient servant,
S. D. GROSS.

—*Phil. Med. Examiner.*

